# Microbiome-producing SCFAs are associated with preterm birth via trophoblast function modulation

**DOI:** 10.1128/mbio.02702-24

**Published:** 2024-11-11

**Authors:** Lulu Meng, Meng Meng, Ruonan Zhang, Ayinisa Wubulikasimu, Hao Peng, Lu Zhang, Xinwen Chang, Guihai Ai, Gang Zou, Qizhi He, Kai Wang, Ming Liu, Tao Duan

**Affiliations:** 1 Department of Obstetrics, Shanghai East Hospital, Tongji University School of Medicine, Shanghai, China; 2 Clinical and Translational Research Center, Shanghai Key Laboratory of Maternal Fetal Medicine, Shanghai Institute of Maternal-Fetal Medicine and Gynecologic Oncology, Shanghai First Maternity and Infant Hospital, School of Medicine, Tongji University, Shanghai, China; 3 Department of Fetal Medicine and Prenatal Diagnosis Center, Shanghai First Maternity and Infant Hospital, Tongji University School of Medicine, Shanghai, China; 4 Department of Obstetrics and Gynecology, Shanghai Tenth People’s Hospital, School of Medicine, Tongji University, Shanghai, China; 5 Department of Pathology, Shanghai First Maternity and Infant Hospital, School of Medicine, Tongji University, Shanghai, China; Columbia University, New York, New York, USA

**Keywords:** 16S rRNA, SCFAs, pregnancy, cervicovaginal microbiota, preterm birth

## Abstract

Although preterm birth (PTB) is one of the major causes of perinatal mortality and neonatal morbidity, little is known about its complex etiology. An abnormal cervicovaginal microbiome during pregnancy is associated with an increased risk of PTB. The cervicovaginal microbiota and its active metabolites, such as short-chain fatty acids (SCFAs), might be effectively used to predict and diagnose PTB. However, the roles of these proteins and the underlying mechanisms involved remain elusive. We conducted 16S rRNA gene sequencing and used a targeted metabolomics approach to study cervicovaginal swabs obtained from 51 singleton pregnancies and 52 twin pregnancies in the second trimester. Next, functional *in vitro* experiments were performed to investigate the roles and mechanisms of SCFAs in placental trophoblast cells (HTR8/SVneo cells). Significant cervicovaginal microbiome dysbiosis, characterized by a substantial reduction in the abundance of lactobacilli and overgrowth of anaerobes, was revealed in the second trimester and was strongly associated with subsequent PTB (*P* = 0.036). Among the paired samples (*n* = 103), acetic acid was significantly greater in the preterm group than in the term group (*P* = 0.047). Data obtained from integrated gas chromatography‒mass spectrometry and 16S RNA studies revealed metabolites that were distinctly associated with particular microbial communities. *Gardnerella vaginalis* was the species most positively associated with acetic acid content. In addition, we identified a marker set consisting of the pregnancy type, acetic acid concentration, and community state type to accurately diagnose PTB. Acetate was associated with increased interleukin (IL)-8 and IL-6 levels and extravillous trophoblast cell migration and invasion through the activation of the extracellular signal-regulated kinase 1/2 signaling pathway in HTR8/SVneo cells. Cervicovaginal microbiota dysbiosis is an important etiological factor of PTB. The cervicovaginal microbiota and its active metabolites can be efficiently used to predict and diagnose PTB. Our findings enrich the microbiota–placenta axis theory and contribute to the development of microecological products for pregnancy.

**IMPORTANCE:**

Preterm birth (PTB) is a leading cause of infant mortality and long-term health issues, affecting millions of families worldwide. Despite its prevalence, the exact causes of PTB remain unclear. Our study reveals that certain bacteria and their metabolic byproducts in the cervicovaginal environment, specifically short-chain fatty acids (SCFAs), are linked to the risk of preterm birth. By analyzing samples from pregnant women, we found that an imbalance in the vaginal microbiota and increased levels of SCFAs are associated with changes in cells that can lead to early labor. This research provides new insights into how the microbiome influences pregnancy outcomes and highlights potential biomarkers for predicting preterm birth. Understanding these microbial influences could lead to innovative strategies for early diagnosis and prevention, ultimately improving maternal and infant health.

## INTRODUCTION

Preterm birth (PTB), defined as birth before 37 weeks of gestation, is a leading cause of perinatal mortality and the second largest cause of death in children under 5 years old worldwide (1). Approximately 15 million preterm babies are born annually, with significant numbers in India and China (2). PTB poses a major public health challenge due to its association with short-term illnesses and long-term complications in children (3). Intrauterine infections, especially ascending bacterial infections from the vagina, are major contributors to PTB, accounting for about 38% of cases (4–9). However, the underlying molecular mechanisms remain unclear, highlighting the need for further research into microbial and molecular interactions in cervicovaginal fluid (CVF).

The vaginal microbiota, primarily consisting of *Lactobacillus* species, plays a crucial role in maintaining a healthy pregnancy by protecting against urogenital infections (10–13). Disruptions in this microbial community, known as vaginal dysbiosis, have been linked to an increased risk of spontaneous PTB (sPTB). The human vaginal microbial communities are classified into five community state types (CSTs), with CST IV, which lacks lactobacilli and is enriched with anaerobic bacteria, being more likely associated with PTB (14–17).

Short-chain fatty acids (SCFAs), metabolites produced by the microbiota, have been implicated in inflammatory responses and may play a role in PTB. SCFAs, metabolites produced by the microbiota, are essential for host metabolic health. SCFAs were detected in both the decidua and the villi (18). To date, numerous studies have demonstrated that the microbiota and their metabolites can induce a local inflammatory response in gestational tissues (acute chorioamnionitis), leading to preterm labor (19–23). In addition, a few studies have investigated the relationship between cervicovaginal microbial communities, metabolic substances, and sPTB (24, 25). An imbalance in the content of SCFAs has been implicated in maternal and fetal health in mouse models (26–29). It remains unclear whether these compounds have biological activity leading to sPTB. Although a few studies have investigated the composition of the vaginal microbiome during pregnancy, limited information is available related to cervicovaginal SCFAs. However, the majority of current studies have assessed the role of the intestinal microbiota. There is limited information on cervicovaginal SCFAs during pregnancy and their potential contribution to PTB. We investigated the cervicovaginal target metabolome (SCFAs) in the second trimester during pregnancy to determine whether it contributed to preterm birth.

We investigate the cervicovaginal microbiota and SCFAs in the second trimester of pregnancy, examining their roles in the pathophysiology of sPTB. By analyzing the microbial communities and their metabolites, we hope to provide new insights into PTB mechanisms and identify potential biomarkers for predicting and preventing this condition.

## MATERIALS AND METHODS

### Study cohort and sample collection

A nested case‒control study was performed in a prospective cohort. The Institutional Review Board approved this study. In total, 103 Chinese women, including 51 with singleton pregnancies and 52 with twin pregnancies, were recruited at the Shanghai First Maternity and Infant Hospital (Fig. S1). Patients with vaginal bleeding, cancer, or endocrine or autoimmune disorders were excluded. Gynecological examination, vaginal drug administration, douching, and sexual activity were restricted within 7 days before the examination (Table 1). Informed consent was obtained from all participants. All sampling was performed by the same physician. The physician swabs into the cervicovaginal site to help surgeons swipe the region of the uterine cervix. The swab was swirled for 30 s to ensure contact with the walls of the vagina so that the swab could absorb cervicovaginal secretions. Nylon flocked swabs (CY-98000PS) from Huachenyang (Shenzhen) Technology Co., Ltd., were used to collect all the samples. The swab heads were divided into frozen pipes with or without microbial preservation solution (BGI-Shenzhen, MGIEasy). The specimens were frozen in liquid nitrogen immediately and stored at −80°C until they were transported on dry ice to BGI-Qingdao for 16S rRNA amplicon sequencing and to Biotree (Shanghai Biotree Biomedical Technology Co., Ltd.) for SCFA analysis. The concentration of SCFAs was determined using a gas chromatography‒mass spectrometry (GC–MS) assay. All cytological tests were normal, and no lesions were detected.

**TABLE 1:** Eligibility and exclusion criteria

Criteria	Factor
Eligibility	Gestational age <23 weeks
	No symptoms of preterm labor
	Intact fetal membranes
Exclusion	Cervical length <25 mm
	Symptoms suggestive of preterm labor PPROM^*a*^
	Genital tract cancer
	Urinary tract infection
	Abnormal cervical cytology
	Recent vaginal examination
	Vaginal bleeding
	Fetal anomaly
	Endocrine/autoimmune disorders
	Vaginal drug administration
	Vaginal douching within 7 days
	Sex life within 7 days

^*a*^ PPROM, preterm prolabor rupture of membranes.

### DNA extraction and 16S rRNA amplicon sequencing

Of sterile phosphate-buffered saline (PBS), 1 mL was added to each frozen pipe, which was rigorously vortexed for 1 min. Next, 500 µL of the solution was collected, centrifuged, and disrupted by enzymatic treatment. The vaginal lavage fluid was centrifuged at 2,000 rpm for 15 min. The total DNA concentration of the samples was determined using a Qubit high-sensitivity kit according to the manufacturer’s instructions. The primers 515F/806R targeting the V4 hypervariable regions of the prokaryotic 16S rRNA gene were used for gene amplification (515F: 5′-GTG CCA GCM GCC GCG GTA A-3′; 806R 5′-GGA CTA CHV GGG TWT CTA AT-3′), followed by DNA extraction. The PCR products were purified and subjected to high-throughput sequencing using an Illumina HiSeq 2,500 at BGI Genomics Co., Ltd. (Shenzhen, China).

### Bioinformatic analysis

16S amplicon analysis was subsequently conducted by OE Biotech Co., Ltd. (Shanghai, China). The raw sequencing data were generated in FASTQ format. Next, paired-end reads were preprocessed using Cutadapt software to detect and cut off the adapter. After trimming, paired-end reads were filtered to remove low-quality sequences, denoised, merged, and detected. The chimeric reads were cut off using DADA2 with the default parameters of QIIME2 (2020.11) (30, 31). Finally, representative reads and an amplicon sequence variant (ASV) abundance table were obtained. The representative read of each ASV was selected using the QIIME2 package. All representative reads were annotated and blasted against the Silva database version 138 using the q2-feature classifier with default parameters. The 16S rRNA V3–4 gene sequence of cervicovaginal bacteria was obtained from 103 samples, from which 78,110 and 86,707 raw reads were produced. The number of clean tags in all the samples ranged between 1,969 and 41,813 reads. Valid reads were obtained between 56,462 and 77,911 after filtering the low-quality raw tags and removing chimeric sequences. ASV analysis revealed a long tail in the rank abundance curves, indicating the low abundance of most ASVs. All the ASVs were evenly distributed (Fig. S2).

### SCFA analysis

Concentrations of SCFAs were measured using gas chromatography‒mass spectrometry (GC2030-QP2020 NX; Shimadzu, Kyoto, Japan) on an Agilent HP-FFAP capillary column (30 m × 250 µm × 0.25 µm; J&W Scientific, Folsom, CA, USA). GC‒MS was performed according to standard protocols by Shanghai Biotree Biomedical Technology Co., Ltd. SCFAs were quantified using the external standard curve method and normalized to the sample weight.

### Cell culture

The HTR-8/SVneo cell line was cultured in Dulbecco’s modified Eagle medium supplemented with 10% fetal bovine serum (FBS, Cyclone) and supplemented with DMEM supplemented with nutrient mixture F-12 medium (Gibco, Life Technologies, Grand Island, NY, USA). The cells were incubated at 37°C with 5% CO_2_ in a humidified incubator.

### EdU incorporation assay

EdU assays were performed using an EdU staining kit (Cat# CX003). The percentage of EdU-positive cells was calculated using the following formula: EdU-positive rate = EdU-positive cell count/(EdU-positive cell count + EdU-negative cell count) × 100%. MGView software was used to count the cells.

### Immunohistochemical staining

Placental tissues were first fixed in 4% buffered formalin and subsequently embedded in paraffin. Tissue sections 3–5 µM in thickness were cut from the paraffin-embedded tissues, mounted on poly-L-lysine-coated slides, deparaffinized in xylene, dehydrated in alcohol, and finally stained with hematoxylin and eosin (H&E). Certain sections were stained for FFAR2, CK-7, and HLA-G using the streptavidin–biotin–horseradish peroxidase complex formation method.

### Immunofluorescence staining

Immunofluorescence staining was performed on frozen tissue slides. The slides were fixed with 4% paraformaldehyde for 15 min and washed thrice with ice-cold PBS. Afterward, the slides were permeabilized with 0.1% Triton X-100 (Thermo Fisher Scientific) for 10 min. Subsequently, the slides were blocked with 5% bovine serum albumin (Thermo Fisher Scientific) for 1 h at room temperature and incubated with primary antibodies against FFAR2 and HLA-G overnight at 4°C. The cells were then incubated with goat anti-rabbit IgG or goat anti-mouse IgG secondary antibodies for 1 h. The stained cells were observed under a confocal microscope (Leica TSC SP8, Mannheim, Germany). The antibodies used are listed in Table S2.

### Cell migration and invasion assays

The invasion and migration of HTR-8/SVneo cells were assessed using Transwell inserts (8 µm, Corning, USA). Matrigel (BD Biosciences)-coated and uncoated membranes were used for invasion and migration assays, respectively. For migration assays, 5 × 10^4^ cells in 0.3 mL of serum-free medium were transferred to the upper chamber. For invasion assays, 10 × 10^4^ cells in 0.3 mL of 2% FBS medium were transferred to the upper chamber. The lower chamber was filled with medium containing 10% FBS. Following incubation at 37°C with 5% CO_2_ and 95% air for 16 h, the fluorescent stain calcein-AM (Thermo Fisher Scientific) was added to each chamber, and the cells were further incubated for 30 min. The number of migrated cells was determined by fluorescence image analysis (Nikon TI-S, Tokyo, Japan).

### Western blot analysis

The concentration of proteins was quantified using the Pierce BCA Protein Assay Kit (WELLBI, WB0123) following the manufacturer’s instructions. The proteins were separated by 10% SDS‒PAGE and transferred to polyvinylidene fluoride membranes by gel electrophoresis and electroblotting. After blocking with 5% bovine serum albumin (BSA), the blots were incubated with primary antibodies at 4°C overnight. Next, the membranes were washed and incubated with secondary antibodies. Ultimately, the protein bands were visualized using enhanced chemiluminescence reagents (Thermo Fisher Scientific). The antibodies used are listed in Table S1. The relative protein expression was analyzed by densitometry using ImageJ software (NIH).

### Enzyme-linked immunosorbent assay

To conduct an enzyme-linked immunosorbent assay (ELISA), the cells were treated with various concentrations of drugs for 24 h. Next, cell-free supernatants were collected, and the concentrations of IL-6, IL-8, and tumor necrosis factor (TNF)-α in the cell culture supernatants were measured using an ELISA kit (Absin) according to the manufacturer’s instructions.

### Statistics

All the data were analyzed using SPSS 26.0 (IBM, Armonk) or Prism 8 software (GraphPad Software, La Jolla, CA, USA). The normality of the data was tested with the Shapiro‒Wilk (S‒W) or Kolmogorov–Smirnov (K–S) test. Unpaired Student’s *t*-tests, Mann‒Whitney *U* tests, or chi-square tests were used where appropriate. Student’s *t*-test was used to compare the results with two means. Ordinary one-way analysis of variance followed by Tukey’s multiple comparison test was used to compare normally distributed data with at least three means. Asterisks denote *P* values as follows: *P  < 0 .05, **P  < 0 .01, and ***P  <  0.001. Other descriptive statistics and tests are shown in the figure legends or tables. Bivariate comparisons of maternal characteristics between cases and controls were performed using the chi-square (χ^2^) test or Student’s *t*-test, as appropriate. Metabolites were analyzed using log2-transformed data normalized to the volume available or utilized for extraction. For metabolites below the limit of detection (LOD), the LOD divided by the square root of 2 was assigned. Student’s *t*-tests were used to compare metabolite abundance between different groups (32).

## RESULTS

### Significant cervical vaginal microbiome dysbiosis in preterm groups

**Fig 1:**
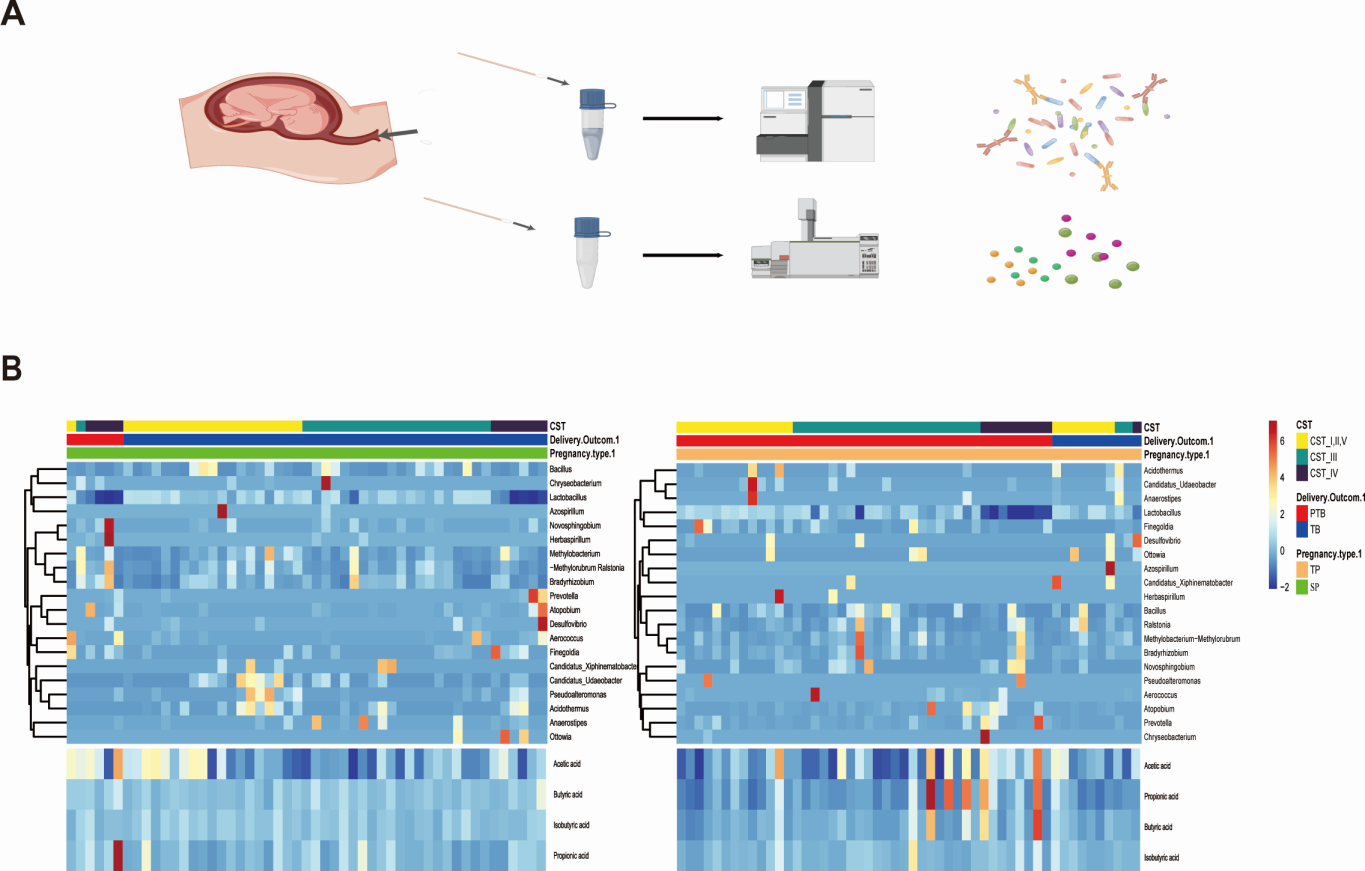
Heatmap of the relative abundance of microbial taxa and four main SCFAs identified in the cervicovaginal microbiota of 103 pregnant women. (**A**) Schematic representation of the study design. (**B**) Ward linkage hierarchical clustering of the Jensen–Shannon metric identified five community state types (CST I, II, III, IV, V). The upper color bar shows the three groups of community state types, while the lower color bar shows each sample’s pregnancy outcome and type (PTB, preterm birth; TB, term birth; TP, twin pregnancy; SP, singleton pregnancy). Colored bars represent the relative abundance of microbial taxa in each replicate sample. The color intensity of the heatmap increases with the taxa’s relative abundance from low (blue) to high (red).

The demographic details of the participants and their corresponding CVF samples are shown in Table 2. Apart from birth outcomes, no other recorded clinical or demographic characteristics differed significantly among the four groups of pregnant women. A total of 103 pregnant women, comprising 52 with twin gestations and 51 with singleton gestations, participated in the study and provided paired samples during the second trimester (gestational age between 12 and 26 weeks) (Fig. S1). The participants were divided into four groups according to pregnancy type and outcome: preterm birth in singleton pregnancies (SP-PTB, *n* = 6), term birth in singleton pregnancies (SP-TB, *n* = 45), preterm birth in twin pregnancies (TP-PTB, *n* = 42), and term birth in twin pregnancies (TP-TB, *n* = 10). CVF analysis provided matched bacterial (16S rRNA gene) and metabolome (SCFA) data sets (Fig. 1A and B). A heatmap revealed different relative abundances of the top 20 genera and four kinds of SCFAs (Fig. 1B).

**TABLE 2:** Descriptive characteristics of the participants^*a*^

Characteristic	Singleton pregnancy (*n* = 51)			Twin pregnancy (*n* = 52)			All (*n* = 103)		
	Term birth >37 weeks (*n* = 45)	Preterm birth <37 weeks (*n* = 6)	*P* value	Term birth >37 weeks (*n* = 10)	Preterm birth <37 weeks (*n* = 42)	*P* value	Term birth >37 weeks (*n* = 55)	Preterm birth <37 weeks (*n* = 48)	*P* value
Maternal age, years	28.07 ± 0.45	28.00 ± 0.82	0.765	29.3 ± 0.88	30.88 ± 0.65	0.239	28.29 ± 3.00	29.52 ± 4.13	0.091
Cervical length, mm	32.26 ± 0.44	31.67 ± 1.17	0.597	32.90 ± 2.47	33.30 ± 1.42	0.834	32.38 ± 4.15	33.10 ± 8.68	0.211
GAAS, weeks	14.22 ± 0.58	13.00 ± 0.63	0.809	18.5 ± 1.85	21.24 ± 0.77		17 ± 4.55	18.21 ± 5.43	0.227
GAAD, weeks	38.97 ± 0.15	29.90 ± 3.16	<0.001^*b*^	37.96 ± 0.25	31.22 ± 0.79	<0.001^*b*^	38.79 ± 1.07	31.06 ± 5.42	0.036^*b*^
Gravidity			0.907			0.1692			0.855
1	19 (42.22%)	2 (33.33%)		3 (30.00%)	21 (50.00%)		22 (40.00%)	23 (47.92%)	
2	12 (26.67%)	2 (33.33%)		1 (10.00%)	9 (21.43%)		13 (23.64%)	11 (22.92%)	
≥3	14 (31.11%)	2 (33.33%)		6 (60.00%)	12 (28.57%)		20 (36.36%)	14 (29.17%)	
Parity			0.331			0.428			0.58
0	35 (77.78%)	6 (100.00%)		6 (60.00%)	32 (76.19%)		41 (74.55%)	38 (79.17%)	
≥1	10 (22.22%)	0		4 (40.00%)	10 (23.81%)		14 (25.45%)	10 (20.83%)	

^*a*^ GAAD, gestational age at delivery; GAAS, gestational age at sampling.

^*b*^ *P* < 0.05.

**Fig 2:**
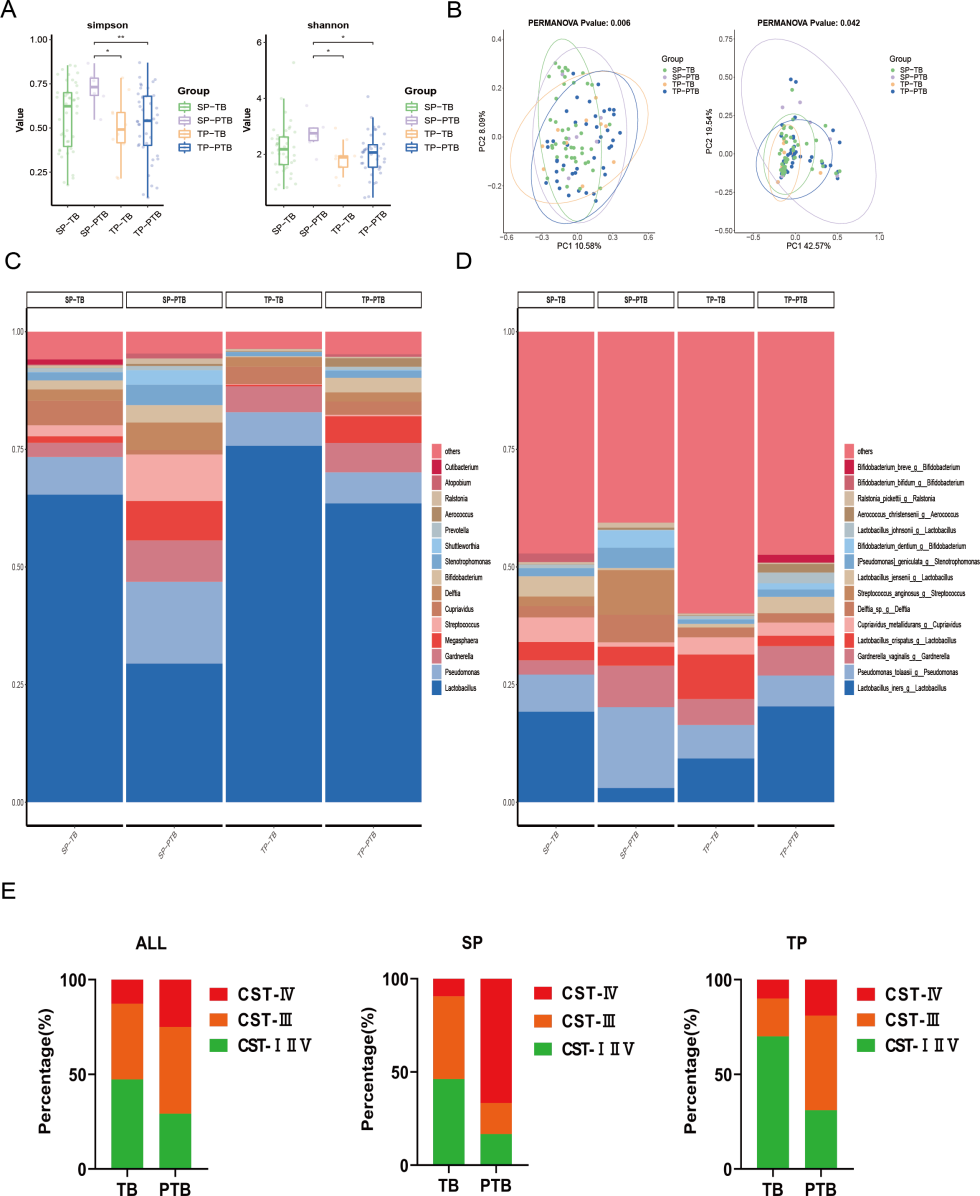
Relationships between CVF microbiota communities and pregnancy outcomes. (**A**) The α diversity of the microbiome was determined by the Simpson index and Shannon index values. *P < 0.05 and **P < 0.01. (**B**) β diversity of the microbiome was determined using PCoA analysis based on unweighted (left) and weighted (right) UniFrac distances. The *P* value corresponding to the PERMANOVA test was less than 0.05. The microbial taxa of the genes and their relative abundance in each group at the genus (**C**) and species (**D**) levels. (**E**) Stacked bar charts showing that the distribution of CSTs differed between TB and PTB patients. *P* for trend <0.05 for linear-by-linear association. PTB, preterm birth; CST, community state type; CST I, *Lactobacillus crispatus*-dominant; CST II, *Lactobacillus gasseri*-dominant; CST III, *Lactobacillus iners*-dominant; CST IV, *Lactobacillus* spp.-depleted; CST V, *Lactobacillus jensenii*-dominant.

Next, we compared the microbial diversity in the CVF among the four groups (Fig. 2A and B). In total, 919 ASVs (74 genera, five phyla) exhibited significant differences among the four groups. Alpha diversity reflected community richness and diversity and was represented by the Simpson estimator and Shannon index, respectively. The difference in the composition of ASVs between PTB patients and healthy controls suggested an increased diversity of microbiomes in PTB patients. The bacterial genus content was greater in PTB patients than in healthy controls. Although the α diversity in both pregnancy types of the PTB group was greater than that in the control group, the differences between the two groups were not significant (*P* = 0.0502, Simpson; *P* = 0.050, Shannon) or in terms of twin pregnancy (*P* = 0.5907, Simpson; *P* = 0.416, Shannon). The α diversity in the SP-PTB group was significantly greater than that in the TP-TB group (*P* = 0.0225, Simpson; *P* = 0.031, Shannon) and the TP-PTB group (*P* = 0.0093, Simpson; *P* = 0.030, Shannon). The same trend was observed when other alpha diversity parameters were analyzed (Fig. S3).

The beta diversity of the SP-PTB group also seemed to be greater than that of the control group. The *P* values were significantly different based on the Adonis test [*P* = 0.042, weighted UniFrac distance; *P* = 0.006, unweighted UniFrac distance; *P* < 0.05, permutational multivariate analysis (PERMANOVA)]. Nonmetric multidimensional scaling ordination revealed a difference in community structure (Fig. S4).

Microbial differences were observed in the CVF for five phyla, 74 genera, and 919 ASVs (Kruskal‒Wallis test). The annotation results revealed that histograms of species in all groups were created for the phylum, class, order, family, genus, and species levels. Histograms showing the relative abundance of species revealed the composition of microbial communities (species and corresponding proportions) in each group at different taxonomic levels. *Firmicutes*, *Proteobacteria*, *Actinobacteriota*, and *Bacteroidota* were the dominant bacterial phyla, accounting for at least 52.62, 14.47, 6.01, and 0.33%, respectively, of the ASVs (Fig. S5A). *Bacilli*, *Gammaproteobacteria*, *Actinobacteria*, and *Negativicutes* were the dominant bacterial classes, accounting for at least 40.35, 14.07, 5.90, and 0.39%, respectively, of the ASVs (Fig. S5B). *Lactobacillales*, *Pseudomonadales*, *Bifidobacteriales*, *and Burkholderiales* were the dominant bacterial orders, accounting for at least 40.01, 6.66, 4.97, and 5.41%, respectively, of the ASVs (Fig. S5C). *Lactobacillaceae*, *Pseudomonadaceae*, *Bifidobacteriaceae*, and *Veillonellaceae* were the dominant bacterial families, accounting for at least 40.01, 6.66, 4.97, and 5.41%, respectively, of the ASVs (Fig. S5C).

*Lactobacillus* was the most abundant genus (average percentage, 58.55%), accounting for 29.49% of the SP-PTB population, 65.41% of the SP-TB population, 63.52% of the TP-PTB population, and 75.78% of the TP-TB population (Fig. 2C). Grouped analysis of the cervicovaginal microbiome revealed that the majority of women in the TB group harbored high levels of *Lactobacillus* in both pregnancy types (≥50%). However, the SP-PTB patients exhibited great heterogeneity, with the lowest percentage of *Lactobacillus* (29.49%), and a few individuals lacked the *Lactobacillus* genus. Different *Lactobacillus* species were distributed in different proportions in the four groups (Fig. 2D).

The bacterial community state types (CSTs) are typically dominated by one of four *Lactobacillus* species: *Lactobacillus crispatus* (CST I), *Lactobacillus gasseri* (CST II), *Lactobacillus iners* (CST III), and *Lactobacillus jensenii* (CST V). However, CST IV lacks *Lactobacillus* and is enriched in several anaerobic bacteria, such as BVAB1, *Gardnerella vaginalis*, *Atopobium vaginae*, and *Prevotella* spp. (14). Hierarchical clustering of the bacterial CSTs revealed that all the samples clustered into five major groups, namely, CST I, CST II, CST III, CST IV, and CST V. Recent evidence suggests that *L. iners* dominance rather than dysbiosis has a high probability of transitioning to higher-risk states (33), and *L. iners*, which is one of the dominant communities, increases the risk of PTBs during pregnancy. *Lactobacillus iners* (CST III) is a vaginal commensal that has recently been demonstrated to be associated with dysbiosis (34). Moreover, NGAL is considered a potential marker of microbial imbalance leading to BV (35). Therefore, we treated CST III (*L. iners*-dominant communities) as a subgroup.

Major differences were detected in the CVF microbial communities at 12 to 26 weeks between women who delivered preterm babies (<37 + 0 weeks) and those who delivered at term (*P =* 0.036; Fig. 2E; Table 3). Stacked bar charts show that the distribution of PTB differs between CSTs (*P =* 0.036; Fig. S6). We compared three distinct populations to determine the effects of CST on PTB incidence. In the second trimester, the predominant CST type of the cervical microbiota was CST III (intermediate type), and the proportion of PTB was 50%. The highest incidence of PTB (63.16%, Fig. S6) was observed among women with the CST IV type. The linear trend of PTB incidence observed in CST status from the protective to intermediate type to the anaerobic type was statistically significant (*P* = 0.036). The composition of the cervicovaginal microbiota in the second trimester was intricately related to PTB incidence, and the difference was more significant for the singleton population (*P =* 0.015; Fig. 1E; Table 3).

**TABLE 3:** Relationships between CVF microbiota communities (CSTs) and PTB among the participants^*a*^

Characteristic	Singleton pregnancy (*n* = 51)			Twin pregnancy (*n* = 52)			All (*n* = 103)		
	Term (*n* = 45)	Preterm (*n* = 6)	*P* value	Term (*n* = 10)	Preterm (*n* = 42)	*P* value	Term (*n* = 55)	Preterm (*n* = 48)	*P* value
CST			0.015^*b*^			0.059			0.036^*b*^
I, II, V	19 (42.2%)	1 (16.7%)		7 (70.0%)	13 (31.0%)		26 (47.3%)	14 (29.2%)	
III	20 (44.4%)	1 (16.7%)		2 (20.0%)	21 (50.0%)		22 (40.0%)	22 (45.8%)	
IV	6 (13.3%)	4 (66.7%)		1 (10.0%)	8 (19.0%)		7 (12.7%)	12 (25%)	

^*a*^ CST, cervicovaginal community state type.

^*b*^ *P* < 0.05.

Next, we identified the microbiological markers of cervicovaginal tissue in the four groups. The relative abundance of the dominant microbes differed at the genus and species levels (Fig. S5E and F). Linear discriminant analysis effect size analysis was performed to determine the ASVs responsible for the differences between sample types to investigate the prognostic microbial markers among the groups (Fig. S7). The greatest differences in taxa among the four communities were detected, and several genera were identified as distinguishing biomarkers (Fig. S7B).

To further reveal the bacterial metabolic pathways and their functional potential, Kyoto Encyclopedia of Genes and Genomes (KEGG) functional orthologs were inferred using the PICRUSt algorithm for 16S-based bacterial members and predicted using the relative abundance of microorganisms in 103 female cervicovaginal swabs (Fig. S8). In total, 46 individual pathways were predicted for singleton (Fig. S8A) and twin (Fig. S8B) pregnancies for preterm and term labor. The PTB group had a lower proportion of genes related to carbohydrate metabolism and a greater proportion of genes related to cell motility and the immune system than the other groups. In addition, KEGG pathway analysis revealed an increased abundance of fatty acid biosynthesis and elongation in the PTB group. Microorganisms colonizing the cervicovaginal microenvironment produce numerous metabolites that promote cervicovaginal health.

Therefore, we investigated whether differences related to fatty acids in the cervicovaginal microbiota, in association with the occurrence of preterm birth, could explain the variations observed in the metabolome data set. Next, we compared the differences in SCFA concentrations between paired cervicovaginal swabs by GC‒MS.

### Significant increase in cervicovaginal acetic acid in preterm birth patients and correlation of SCFAs with the microbiome

**Fig 3:**
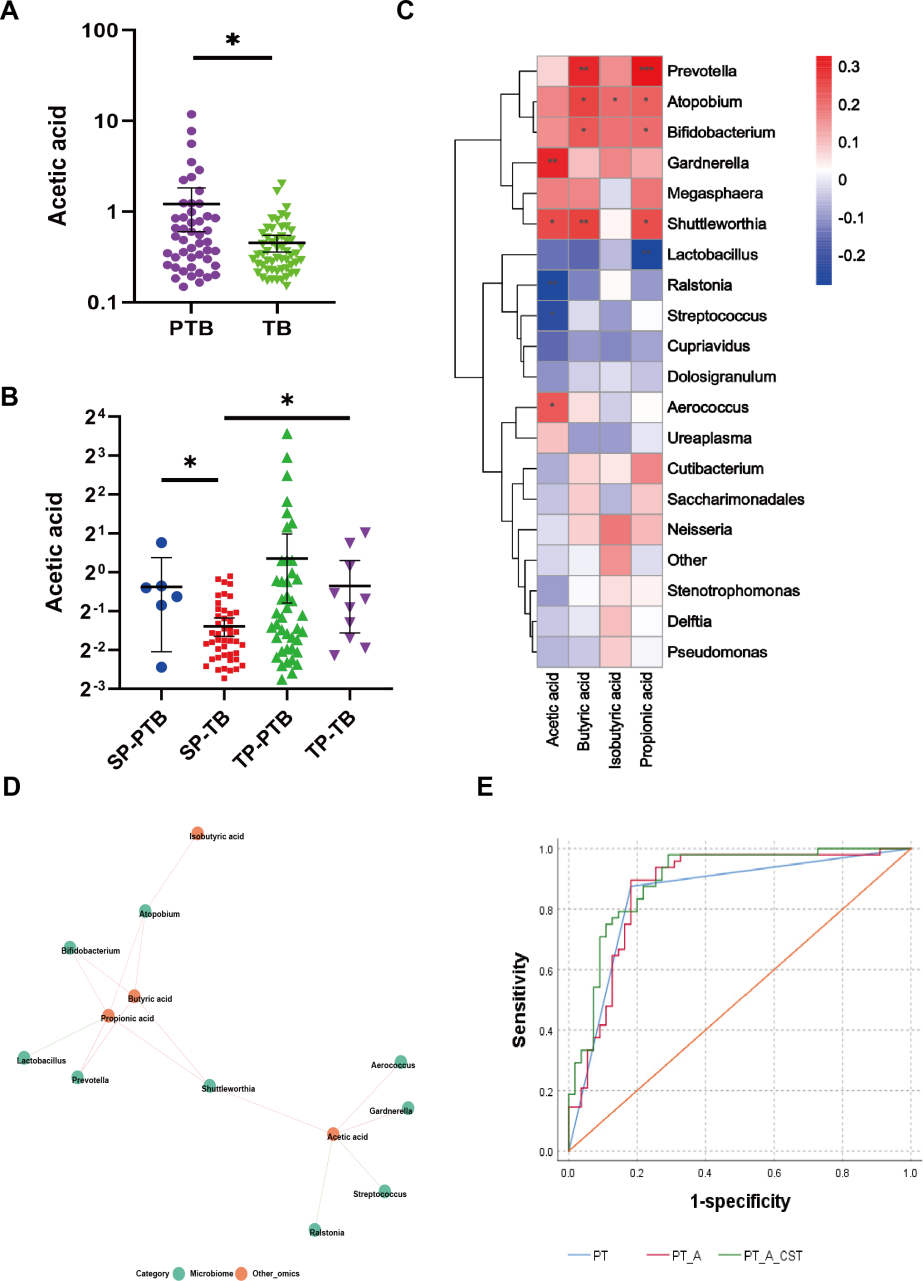
Correlation analysis of taxa and metabolites. (**A**) The acetic acid concentration in the preterm group was significantly greater than that in the term group (*n* = 103, *P* = 0.0152). (**B**) SP-PTB vs. SP-TB, *P* = 0.0341; SP-TB vs. TP-TB, *P* = 0.0208. (**C**) Heatmap showing Spearman’s correlation between metabolites and microbiota composition at the genus level. Asterisks indicate a statistically significant correlation at the level of (*: *P* value < 0.05; **: *P* value < 0.01; ***: *P* value < 0.001), and the colors denote positive (red) and negative (blue) correlation values. (**D**) Network plot highlighting the highly correlated metabolites and microbiota. The figure shows the network diagram based on Spearman correlation analysis to calculate the correlation between species and metabolite data, and the relationship pairs with *P* values < 0.05 were selected. The red line indicates a positive correlation, and the green line indicates a negative correlation. The thickness of the line indicates the correlation coefficient. (**E**) Receiver operating characteristic (ROC) curve areas and AUC for predicting spontaneous preterm birth in patients in the composite models. PT, pregnancy type; A, acetic acid.

The concentration of CVF acetic acid was significantly greater in the PTB group (*P =* 0.047; Fig. 3A). Eleven acids were identified, including acetic acid, propionic acid, isobutyric acid, butyric acid, isovaleric acid, valeric acid, hexanoic acid, heptanoic acid, octanoic acid, nonanoic acid, and decanoic acid. No significant differences were observed in CVF SCFA concentrations between the TB and PTB groups, except for acetic acid (Table S3). Although the concentration of acetic acid in both pregnancy types of the PTB group was higher than in the TB group, the difference between the two groups in singleton pregnancy was statistically significant (*P =* 0.012, Fig. 3B).

Spearman’s correlation analysis was also conducted to assess the relationship between the microbiota and SCFAs (Fig. 3C). SCFAs were negatively correlated with *Lactobacillus* and positively correlated with *Prevotella*, *Atopobium*, *Bifidobacterium*, and *Gardnerella*. The dot plot network illustrates the relationships between the highly correlated SCFAs and the microbiota at the genus level. Acetic acid was negatively correlated with *Gardnerella*, *Shuttleworthia*, and *Aerococcus* at the genus level (*P* < 0.05; Fig. 3D). *Gardnerella vaginalis* (*P* < 0.01) and *Aerococcus christensenii* (*P* < 0.05) were the most positively correlated with acetic acid, whereas *L. iners* was most negatively correlated with acetic acid at the species level (*P* < 0.05; Fig. S9).

### Diagnostic potential of cervicovaginal microbiota communities and acetic acid for preterm birth diagnosis

The content of acetic acid in the CVF was significantly greater in the CST IV group than in the other group (Kruskal–Wallis test, *: *P* < 0.01; Table 4).

**TABLE 4:** Correlation analysis between microbiota status and SCFAs^*a*^

SCFA	CST			*P* value
	I, II, V	III	IV	
Acetic acid	0.722 (0.334, 1.105)	0.611 (0.316, 0.906)	1.443 (0.177, 2.709)	0.002^*b*^
Propionic acid	0.026 (0.021, 0.031)	0.105 (0.001, 0.210)	0.106 (0.010, 0.202)	0.064
Isobutyric acid	0.011 (0.010, 0.013)	0.012 (0.009, 0.015)	0.012 (0.009, 0.015)	0.920
Butyric acid	0.013 (0.011, 0.014)	0.017 (0.010, 0.024)	0.044 (0.010, 0.098)	0.437

^*a*^ Kruskal‒Wallis test.

^*b*^ *P* < 0.01.

The receiver operating characteristic (ROC) curve was generated, and the corresponding area under the curve (AUC) was calculated. Individually, only pregnancy type (AUC = 0.847, 95% CI = 0.766, 0.927) was predictive of PTB (Fig. 3E; Table 5). However, two multivariable models accurately predicting PTB were also identified. The first model included a combination of pregnancy type and acetic acid (AUC = 0.870, 95% CI = 0.797–0.943), and the second model included pregnancy type, CST, and acetic acid (AUC = 0.895, 95% CI = 0.832–0.957) (Fig. 3E; Table 5).

**TABLE 5:** Combined predictive values of pregnancy type, CVF, acetic acid concentration, and CST for preterm birth^*a*^

Test	AUROC (95% CI)
Pregnancy type	0.847 (0.766, 0.927)
Pregnancy type + acetic acid	0.870 (0.797, 0.943)
Pregnancy type + CSTs + acetic acid	0.895 (0.832, 0.957)

^*a*^ AUROC, area under the ROC curve.

### Expression and distribution of FFAR2 in human placental tissues

**Fig 4:**
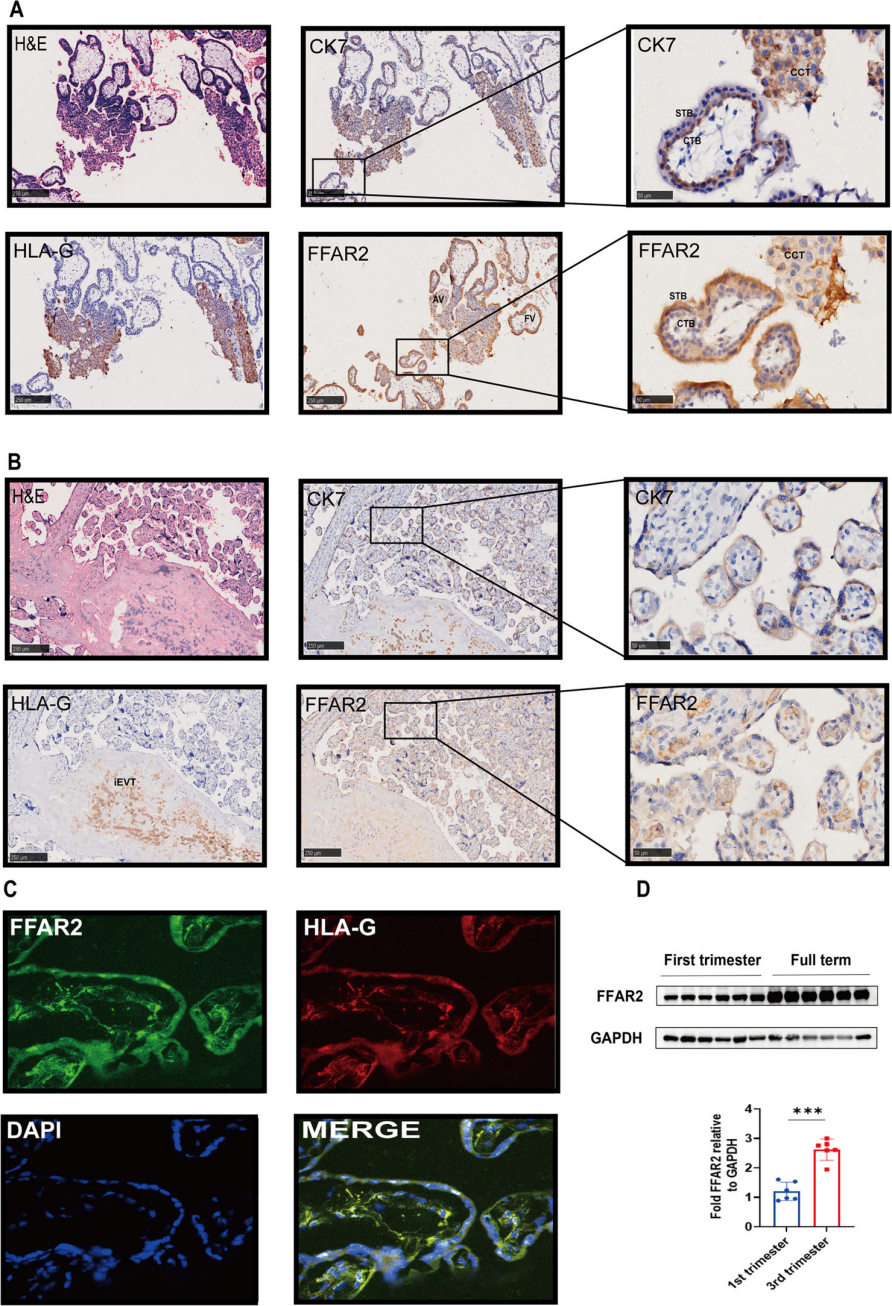
Expression and distribution of FFAR2 in placental tissue. (**A**) H&E staining and immunolocalization of HLA-G, CK7, and FFAR2 in human placental villi from pregnant women (bar, 250 µm/50 µm). (**B**) HE staining and immunolocalization of HLA-G, CK7, and FFAR2 in full-term human placentas from normal pregnancies. (Bar, 250 µm/50 µm). The brown color indicates positive staining for HLA-G, CK7, and FFAR2. CCT, cell column trophoblast; STB, syncytiotrophoblast; CTB, cytotrophoblast. (**C**) Immunofluorescence detection of FFAR2 (green) and HLA-G (red) in the placenta of first trimester. Nuclei were counterstained with DAPI (blue). Merge, merge of FFAR2, HLA-G, and DAPI. (**D**) Western blotting of FFAR2 in first-trimester human villi and full-term placentas from normal pregnancies. Representative Western blot images are shown for FFAR2 and GAPDH. ***P < 0.001 indicates that the difference between the two groups was statistically significant.

To evaluate the function of acetic acid in placental trophoblasts, we first determined the location and expression of the acetic acid functional receptor FFAR2 protein in the placenta by immunohistochemistry, immunofluorescence, and western blotting. Our data demonstrated that FFAR2 was expressed in the villous syncytiotrophoblast layer (STB), cytotrophoblast layer (CTB), and cell column trophoblast (CCT) of the placenta throughout gestation (Fig. 4A and B). FFAR2 is abundantly expressed in migratory extravillous trophoblast cells (EVTs) of the distal anchoring column and invasive EVTs in the maternal endometrium (Fig. 4A). HLA-G is a selective marker expressed by EVT cells (36). Immunofluorescence co-staining for FFAR2 and HLA-G (Fig. 4C) revealed that the expression of FFAR2 increased with gestational age, as shown by western blotting. Moreover, high levels of FFAR2 were observed in placental tissues beginning in the third trimester (Fig. 4D). Taken together, these findings suggest the strong possibility that FFAR2 is involved in regulating trophoblasts and, in particular, EVT cell migration and invasion functions.

### Acetate increases inflammation and promotes trophoblast migration by activating the ERK pathway

**Fig 5:**
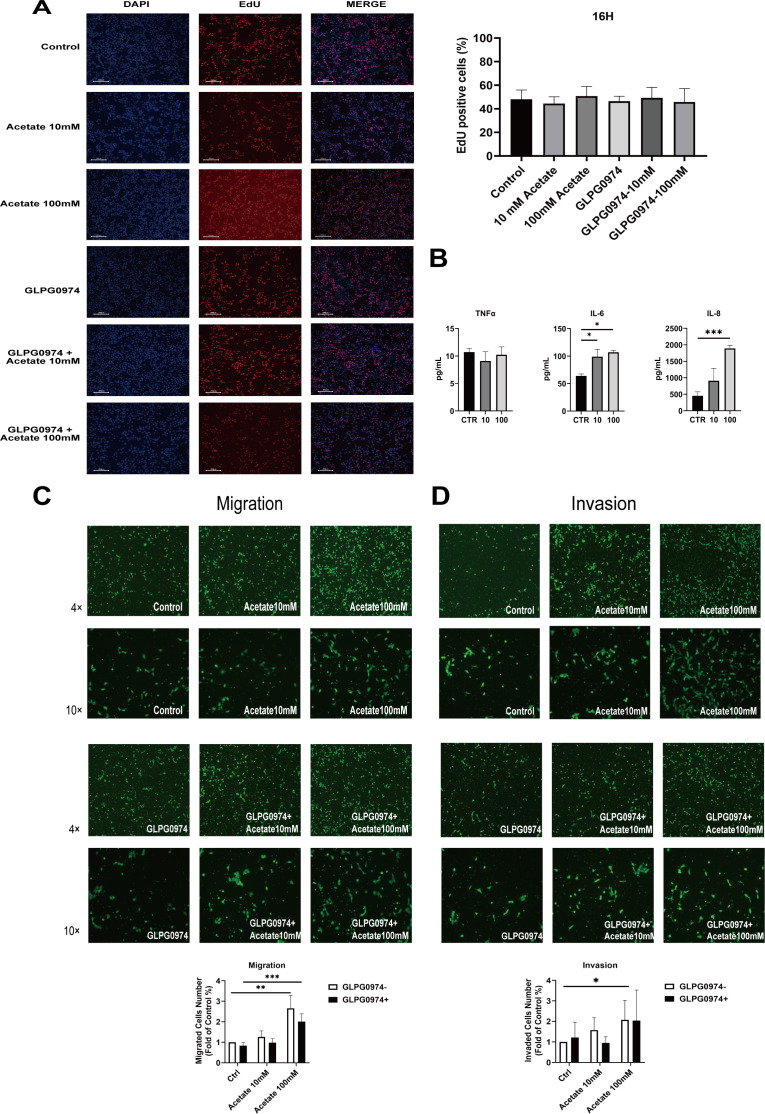
Effects of acetate on HTR-8/SVneo cells. (**A**) Growth analysis of HTR-8/SVneo cells was performed after treatment with acetate for 16 h. EdU (pink) staining of HTR-8/SVneo cells treated with various concentrations of acetate (10 mM, 100 mM). Nuclei were counterstained with DAPI (blue). Scale bars, 200 mm. (**B**) Acetate induces inflammatory cytokine secretion in HTR8/SVneo cells. Migration (**C**) and invasion (**D**) were analyzed in HTR8/SVneo cells with or without acetate treatment. Migration (**C**) and invasion (**D**) were analyzed in HTR8/SVneo cells inhibited by treatment with GLPG0974, a FFAR2 inhibitor. Four fields of view were examined, and the number of cells in the field of view was calculated. Four fields of view were examined, and the number of cells in the field of view was calculated. The cell numbers are presented as the means ± SEM of 3 independent experiments performed in triplicate. (one-way ANOVA, *P < 0.05, **P < 0.01, ***P < 0.001).

Because the function of trophoblasts expressing FFAR2 and acetate could be dependent on these cells and on FFAR2, we next assessed the effects of acetate on a placental trophoblastic cell line (HTR8/SVneo cells). The HTR-8/SVneo cell line, the most widely used trophoblast cell line, was used to determine the effect of acetate on cell proliferation, secretion, migration, and invasion because appropriate trophoblast function is critical for normal placental development and pregnancy maintenance. The concentrations used were 0 and 10 to 100 mM, similar to those used in a previous *in vitro* study (37). EdU assays were performed to evaluate the effects of acetate on the proliferation of HTR8/SVneo cells. Our data indicated that acetate had no effect on cell proliferation. EdU assays revealed no significant decrease in the proliferation of HTR8/SVneo cells treated with acetate compared with that of control cells (Fig. 5A).

PTB has been reported to be associated with a vaginal cytokine profile comprising pro-inflammatory cytokines, including IL-1β and IL-6 (38). Chronic dysregulation of inflammatory responses results in a systemic pro-inflammatory state characterized by elevated levels of cytokines such as interleukin IL-1, IL-6, IL-8, and TNF-α (39). IL-1 and IL-6 have been reported to be the most significant contributors to PTB occurrence. HTR8/SVneo cells were treated with varying concentrations of acetate for 24 h, and cell-free supernatants were collected for cytokine (IL-6, IL-8, and TNF-α) analysis via ELISA. The levels of IL-6 (*P* < 0.05) and IL-8 (100 mM, *P* < 0.001) were significantly increased; however, no significant change in TNF-α levels was observed (Fig. 5B).

In addition, we performed transwell assays to investigate the effects of acetate on trophoblast cell migration and invasion. We found that 10 mM acetate had no effect on cell migration or invasion. However, compared with the control, 100 mM acetate significantly increased the migration (Fig. 5C) and invasion (Fig. 5D) of HTR-8/SVneo cells. The FFAR2 inhibitor GLPG0974 attenuated the migration-promoting effect of acetate (Fig. 5C and D).

**Fig 6:**
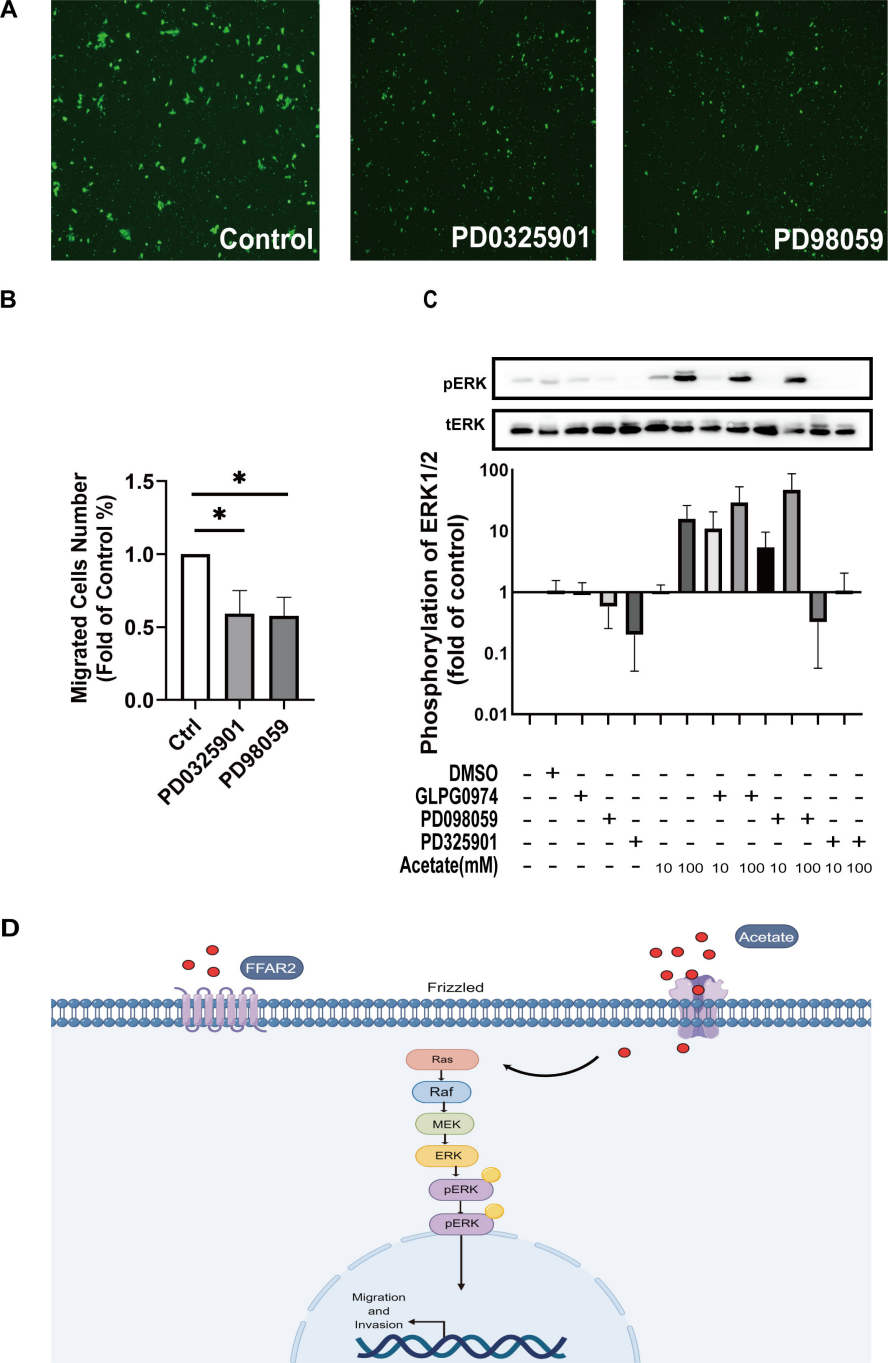
The ERK signaling pathway is implicated in migration and invasion through the induction of acetate. (A) The ERK inhibitor (PD98059/PD0325901) inhibited HTR8/SVneo cell migration after pretreatment for 1 h. (B) Four fields of view were examined, and the number of cells in the field of view was calculated. The cell numbers are presented as the means ± SEM of 3 independent experiments performed in triplicate. (one-way ANOVA, *P < 0.05). (C) WB analysis of total and phospho-ERK1/2 levels in trophoblast cell lines. (D) Cartoon plot showing the mechanism by which acetate promotes the migration of HTR8/SVneo cells through the ERK pathway.

We next investigated the mechanisms contributing to the enhanced signaling in the ERK pathway in HTR-8/SVneo cells. PD98059/PD0325901, an ERK inhibitor, significantly inhibited the migration of cells (*P* < 0.05; Fig. 6A and B). In addition, it inhibited the acetate–FFAR2-induced phosphorylation of ERK (Fig. 6C). The ERK signaling pathway is involved in cell proliferation, differentiation, cytoskeleton reorganization, and cell migration. Previous reports have demonstrated that the ERK signaling pathway is one of the major signaling pathways upstream of FFAR2. PD98059 significantly decreased ERK2 phosphorylation, suggesting that PD98059 effectively blocked the MAPK pathway. A schematic representation demonstrating the mechanism by which acetate promotes the migration of HTR8/SVneo cells through the ERK pathway is shown in Fig. 6D.

## DISCUSSION

We investigated the second-trimester cervicovaginal microbiome and target metabolome of 103 pregnant women in this study. Our results revealed an association between dysbacteriosis-related communities and subsequent sPTB delivery. We further investigated the intriguing interactions between metabolites and potential microbes and demonstrated a strong link between altered acetic acid metabolism and sPTB across the cohort. Furthermore, we used microbiome–metabolome combined models to predict subsequent sPTB weeks to months in advance, contributing to early diagnosis and prevention of PTBs. Acetate increased the levels of IL-8 and IL-6 and promoted the migration and invasion of HTR8/SVneo cells by activating ERK signaling. In addition, our results highlighted that SCFAs are strongly associated with pregnancy, suggesting that they are important impact factors.

The complex pathophysiology of sPTB is ascribed to disturbances in the cervicovaginal microbiota. However, its clinical applications have yet to be realized, and limited consideration has been given to the contribution of the vaginal microbiota profile to the development of sPTB. One proposed mechanism is that the metabolic byproducts of several bacterial species in the microbiome can modulate inflammatory responses in the cervix and uterus, leading to PTB (25). To date, only a few studies have examined the relationship between cervicovaginal microbial communities, metabolic substances, and sPTB (24, 25, 40). SCFAs have also been implicated in pathogenic infections of reproductive issues (40, 41). SCFAs are known to regulate multiple biological processes, including immunity and inflammation, and are thus plausible contributors to PTB. However, the role of these genes in PTB has largely not been explored, especially with respect to human studies. Most related research has been conducted in animal models or *in vitro* systems (26–29), and the majority of related studies have focused on the intestinal microbiota. Our results indicated that an imbalance in the abundance of the vaginal microbiome during midpregnancy is associated with an increased risk of PTB. Acetic acid could serve as a potential biomarker. Our results support previous research indicating that a combination of a dysbiotic vaginal microbiome and high concentrations of acetic acid is a key predictor of sPTB.

We studied the microbiota and metabolites (SCFAs) of cervicovaginal secretions from singleton and twin pregnancies during midpregnancy using high-throughput sequencing methods to examine cervicovaginal fluid samples. Decreased taxon abundance and reduced diversity of CST in twin pregnancies suggest that microbiome alterations are additional factors, if not causal factors, in the multifactorial pathway leading to an increased risk of PTB and worse neonatal outcomes in twin pregnancies (42–44). The differences observed between twin and singleton pregnancies in this pilot study warrant further studies to elucidate the underlying mechanisms involved, particularly longitudinal microbiome sampling in racially and ethnically diverse populations and the context of the short cervix and PTB. A broader understanding of these concepts could help improve risk stratification for PTB in twins. CST III has been reported to switch to CST IV (33). CST III promotes microbiome perturbations by increasing the vaginal pH and producing species-specific virulence factors (45, 46). However, the potential role of these genes in the pathogenesis of PTB requires further investigation. *L. crispatus*, which predominantly produces LA, is a more favorable commensal *Lactobacillus* sp. due to its greater stability and association with lower levels of genital inflammation and sexually transmitted disease-related acquisition. Cultures of *L. crispatus* and *L. jensenii* have been reported to produce substantially more LA than *L. iners* culture. One limitation of this study is the relatively small sample size, which may restrict the generalizability of the findings.

Previous studies have suggested an association between SCFAs and the activation of pro-inflammatory pathways (47). Furthermore, high concentrations of acetic acid were detected in vaginal fluid from women with BV and in culture supernatants from the genera *Prevotella* and *Mobiluncus* (48). Although several metabolites may also contribute to PTB (49), our data suggest that acetic acid is a major bioactive component contributing to this effect. These findings broadly support the findings of other studies in this area linking acetic acid with delivery outcomes (50–53). *Aerococcus* spp. have been linked to acetic acid content, can infect damaged tissues (54), and are associated with risk factors, such as granulocytopenia, prolonged hospitalization, and previous antibiotic treatment (55). It has been implicated in severe polymicrobial chorioamnionitis and subacute bacterial endocarditis (56–59). Acetates are the most abundant SCFAs present in the portal and peripheral blood (60). This ecological dysregulation directly affects the production of pathogenic microbiota metabolites by altering the vaginal pH and inducing the release of pro-inflammatory cytokines (IL-6, IL-8, etc.) and immune cells responsible for PTB (61). Therefore, we used a reductionist approach to examine the specific effects of acetic acid added directly to the cell culture media on placental trophoblasts. We showed that acetate exerts concentration-dependent migration- and invasion-promoting effects on HTR-8/SVneo cells. However, a few studies have implicated propionate in trophoblastic invasion (28). Recent evidence shows that SCFAs can modify the epigenome and affect tissues and organs beyond the gut, affecting health and producing a diseased state; however, whether trophoblast cells can express SCFA functional receptors or transporters requires further investigation. Our study provides perspectives for understanding the potential activities and functions of microbially associated SCFAs, as well as the possibilities for harnessing them to promote personalized and precision therapeutic interventions.

### Conclusion

In summary, our data revealed the associations of differential cervicovaginal microbiota communities and their metabolites with PTB. In addition, we investigated the effects of acetic acid on placental trophoblast function *in vitro*, with important implications for PTB prevention. We believe the findings of this study will provide a better understanding of the role of SCFAs in placental growth and development during pregnancy and highlight the potential of FFAR2 as a therapeutic target for placental disorders in the future.

## Data Availability

Microbiota sequencing data in this study are available from the National Center for Biotechnology Information (NCBI) under accession number PRJNA1177804.

## SUPPLEMENTAL MATERIAL

Supplemental figures mbio.02702-24-s0001.doc Figures S1 to S9.

Supplemental tables mbio.02702-24-s0002.docx Tables S1 to S3.

ASM does not own the copyrights to Supplemental Material that may be linked to, or accessed through, an article. The authors have granted ASM a non-exclusive, world-wide license to publish the Supplemental Material files. Please contact the corresponding author directly for reuse.
