# Supplementary material for: Microbiome-producing SCFAs are associated with preterm birth via trophoblast function modulation
Source: mBio. 2024 Nov 11;15(12):e02702-24. doi: 10.1128/mbio.02702-24 (PMC11633107; doi:10.1128/mbio.02702-24)
Supplement: Supplemental figures — Figures S1 to S9. [file mbio.02702-24-s0001.doc]

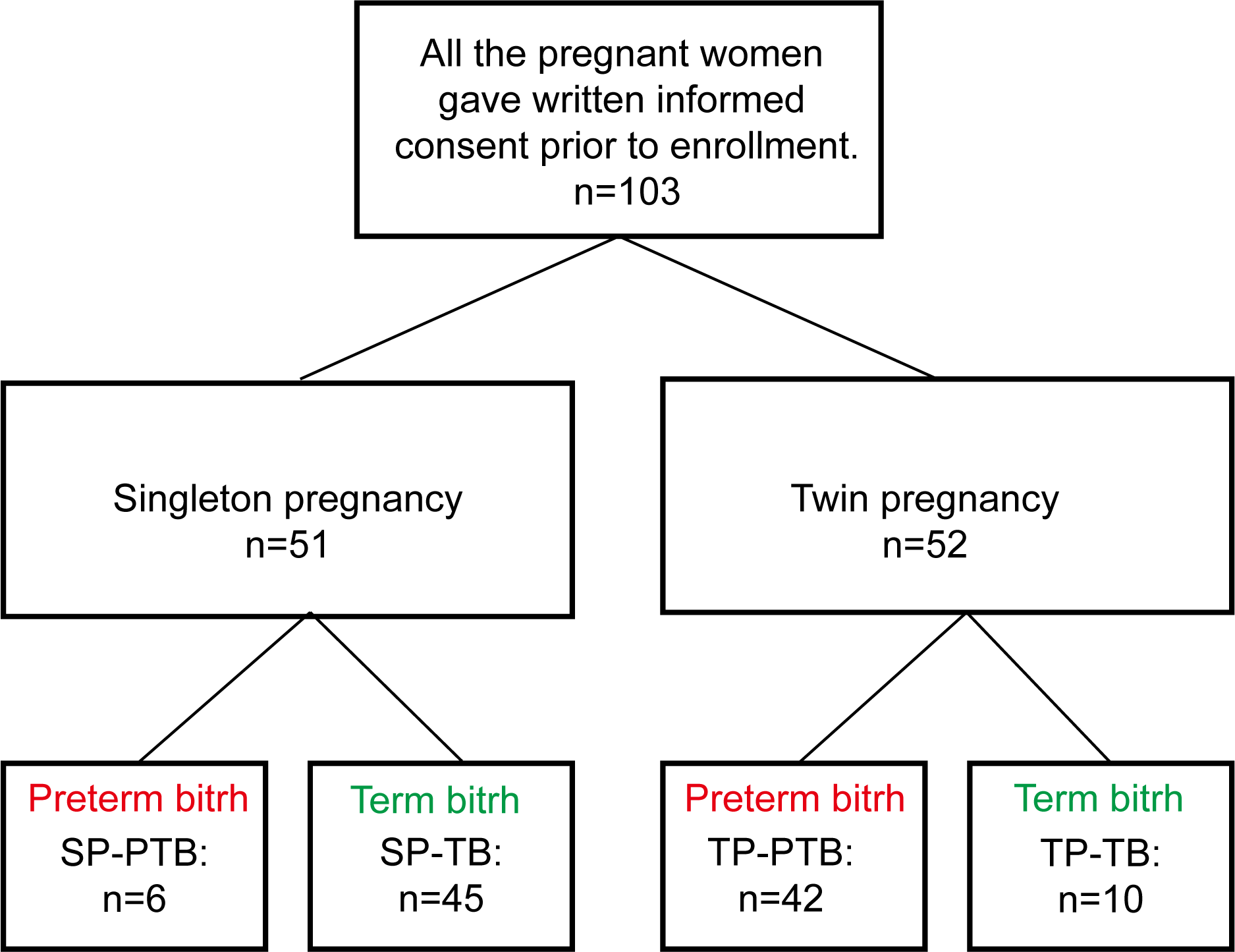


**Fig. S1 Flow chart of the study population.** Based on medical examinations, pregnant women (n = 103) were allocated into the singleton pregnancy group (n =51) and twin pregnancy group (n = 52).


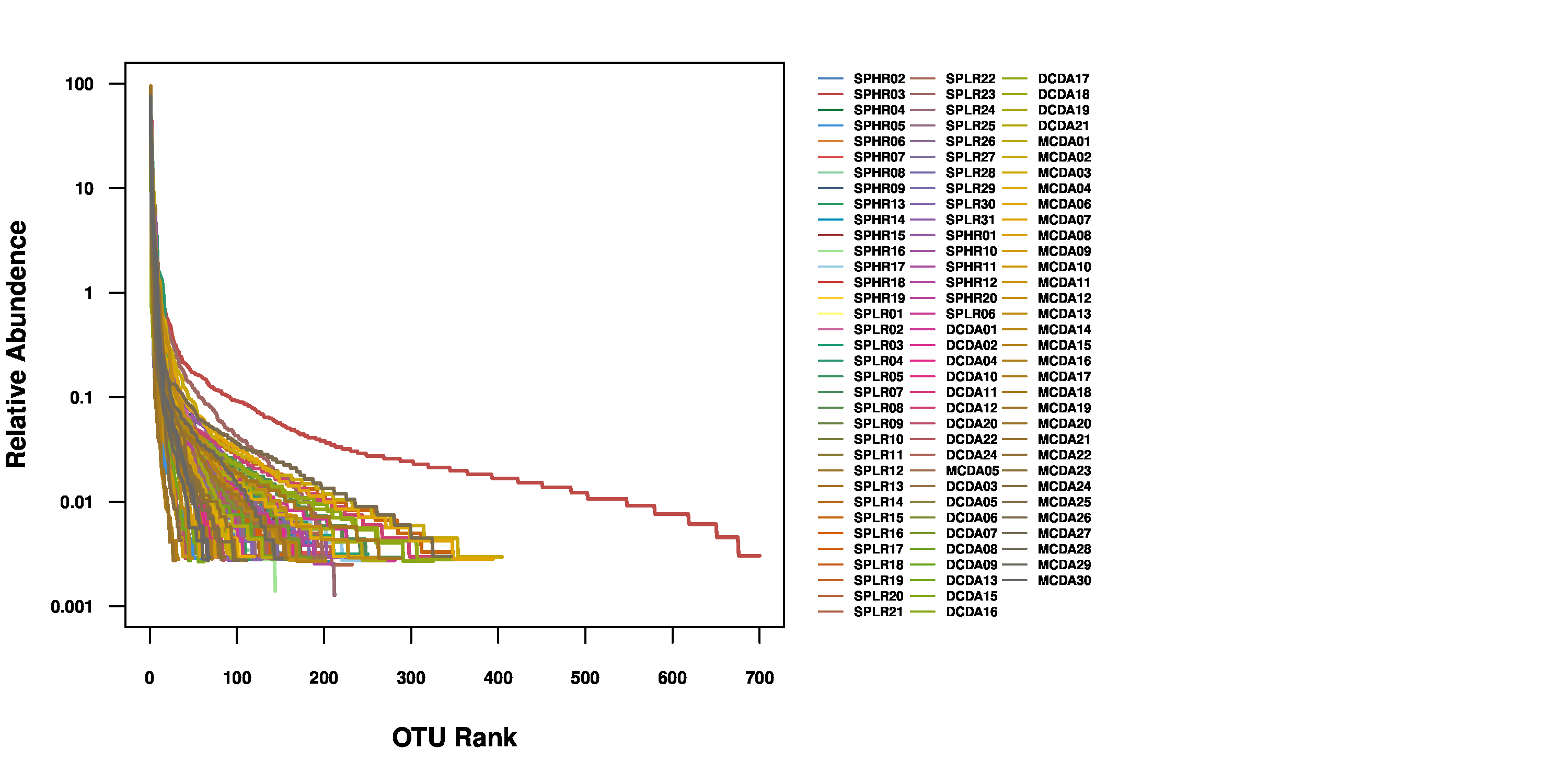


**Fig. S2** OTU level Rank-Abundance curves.


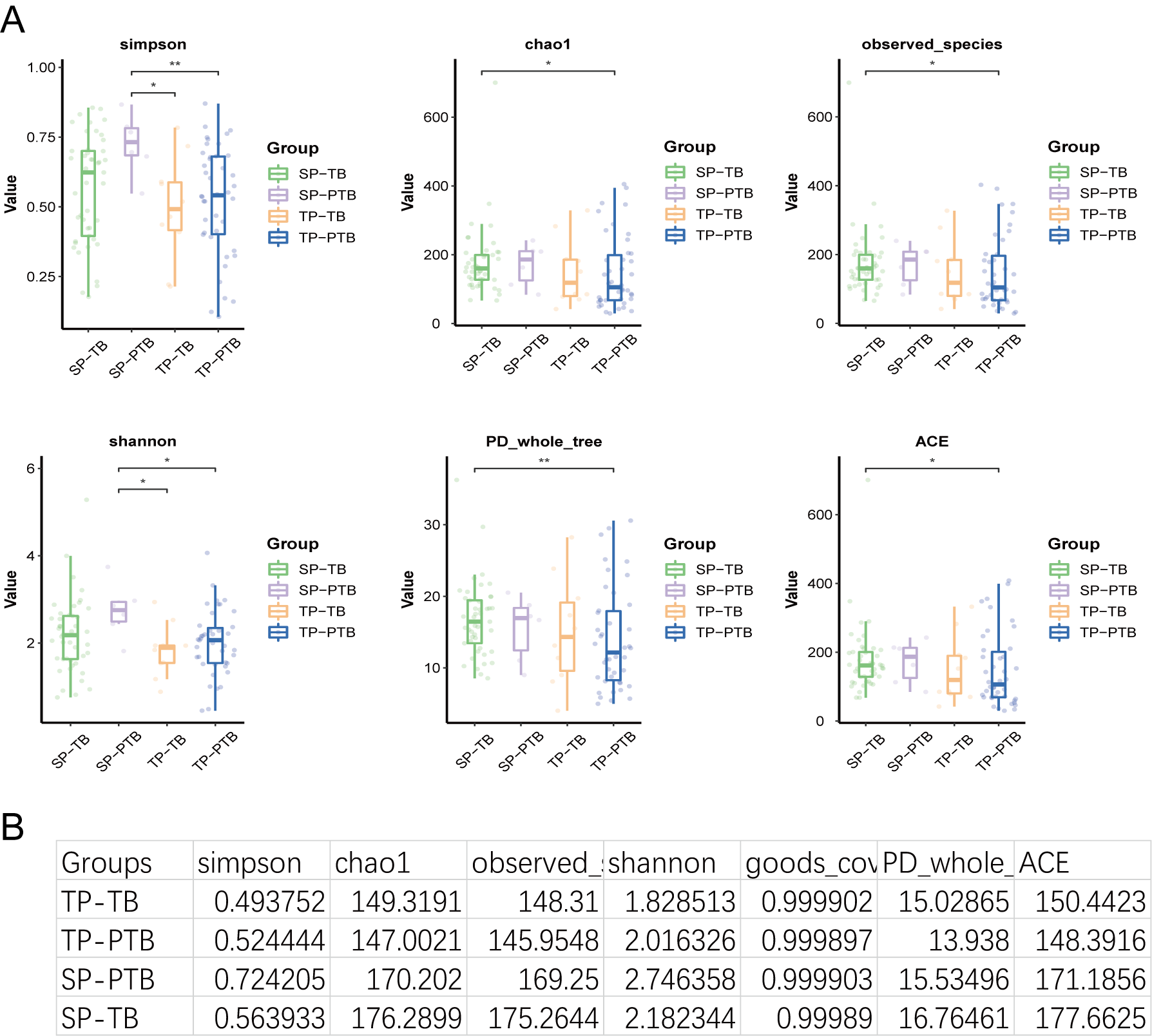


**Fig. S3 Alpha diversity estimator summary.**


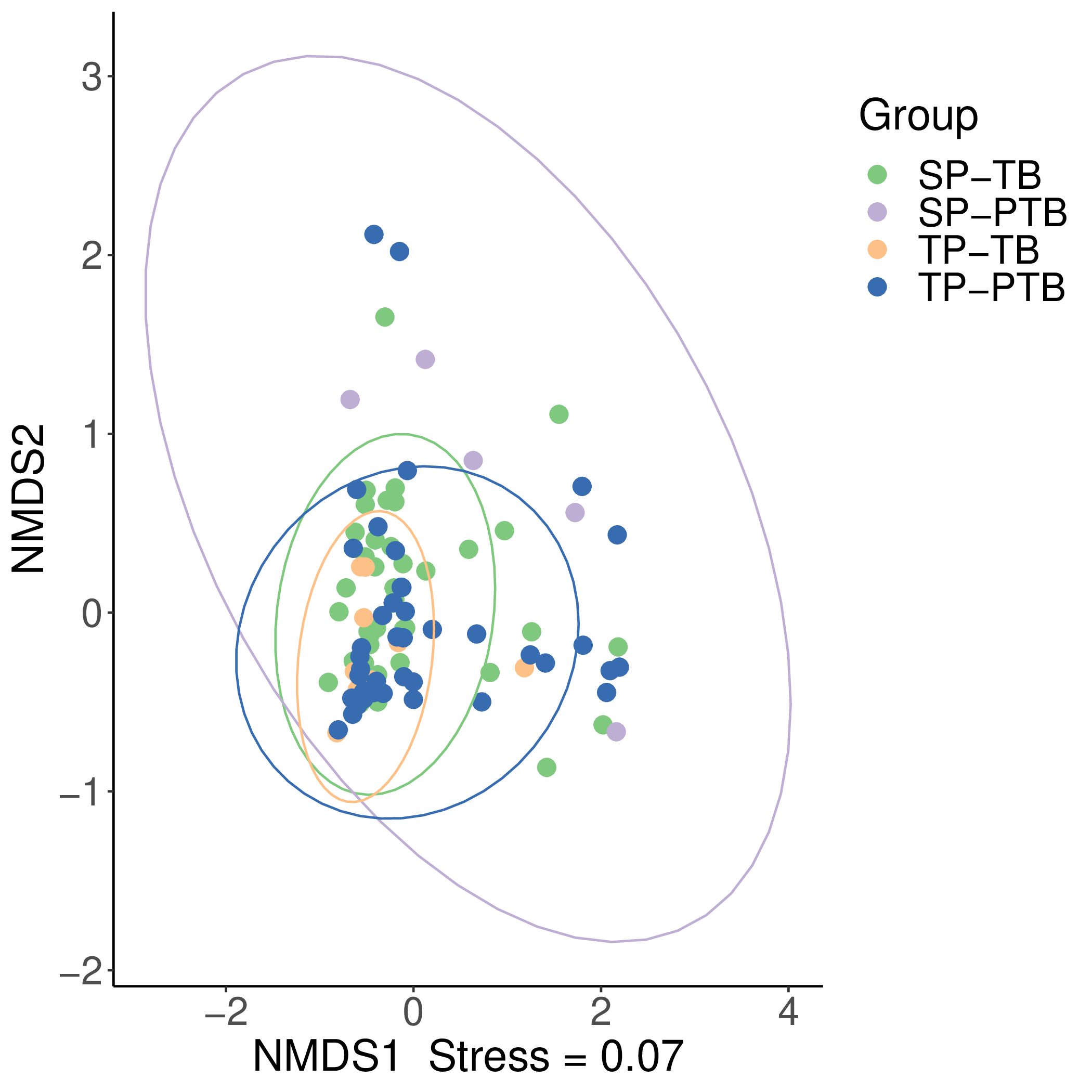


**Fig. S4** Comparing sample distribution belonging to different grs by using weighted non-metric multidimensional scaling (NMDS) analysis. The stress value of the NMDS was 0.07. Each sample is represented by a dot.


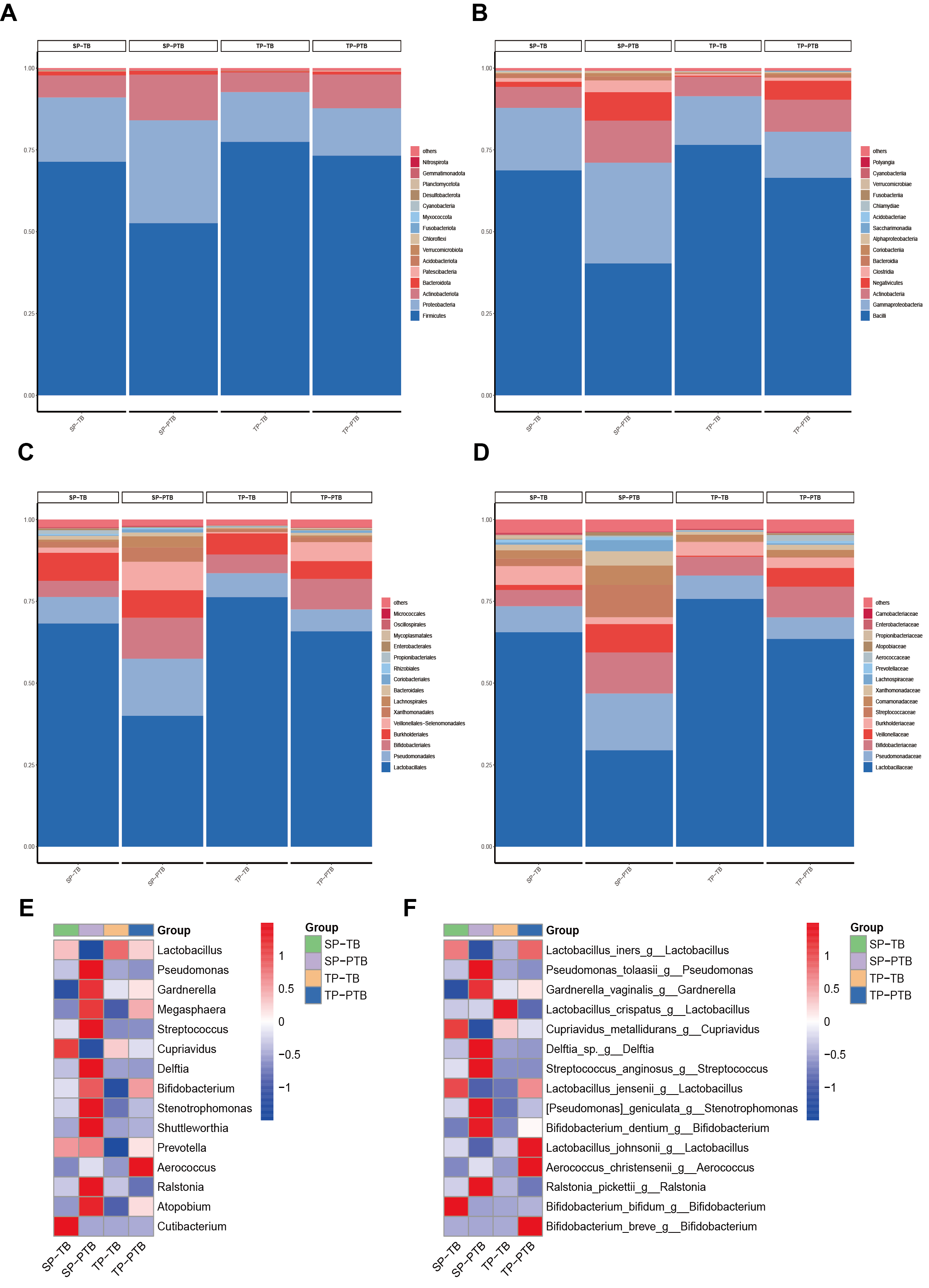
**Fig. S5**  The microbial taxa of the genes and its relative abundance in each group at the Phylum (A), Class (B), Order (C), Family (D) levels. Taxa heatmap at Genus (E), and Species (F) levels in each group. Color intensity of the heatmap increases with the taxa relative abundance from low (blue) to high (red).


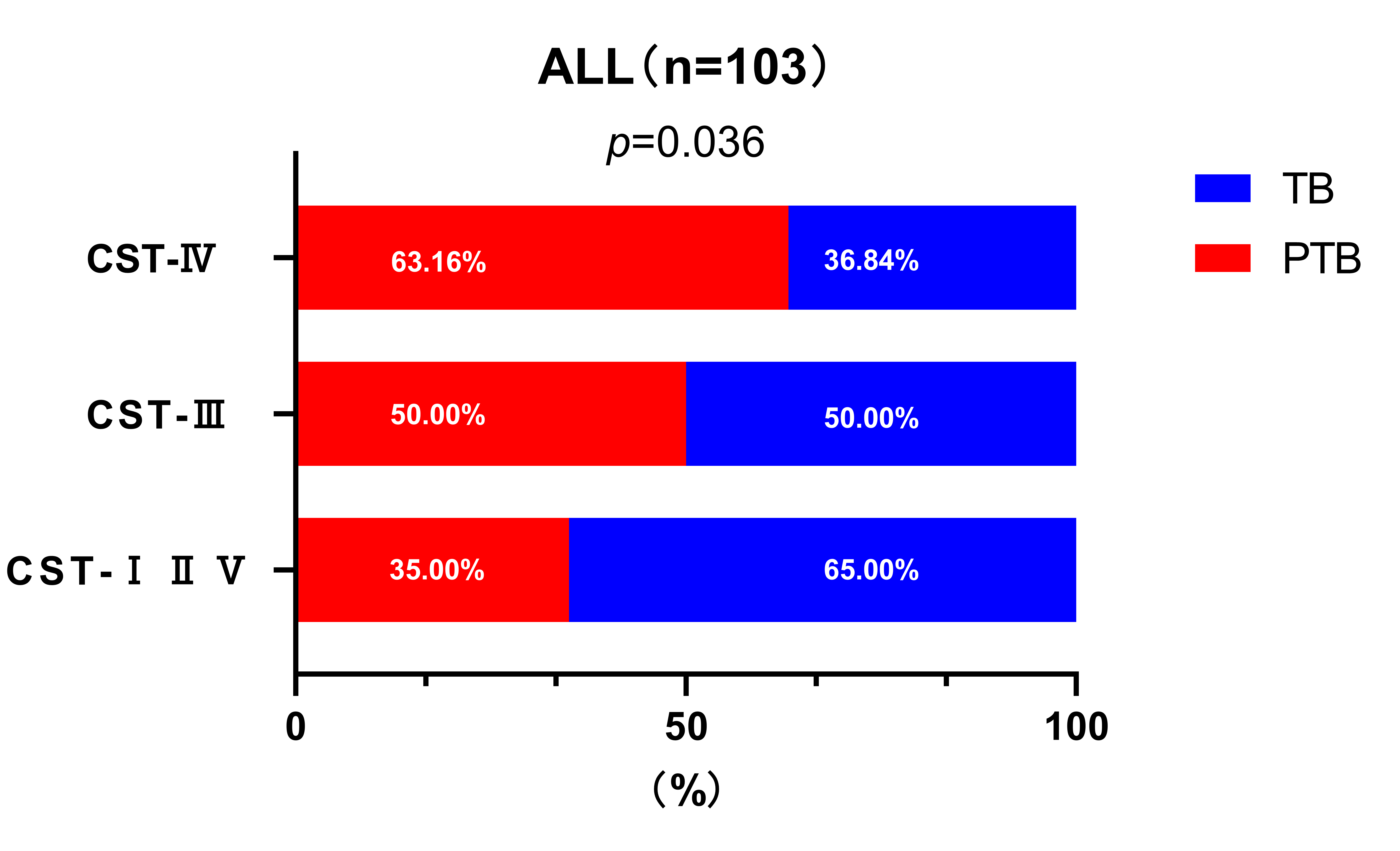


**Fig. S6** **Stacked bar charts show the distribution of PTB differs among CSTs.**

P for trend ＜0.05 in linear-by-linear association. PTB, preterm birth; CST, Community state type; CST I, Lactobacillus crispatus-dominant; CST II, Lactobacillus gasseri-dominant; CST III, Lactobacillus iners-dominant; CST IV: Lactobacillus spp.-depleted; CST V, Lactobacillus jensenii- dominant.

**
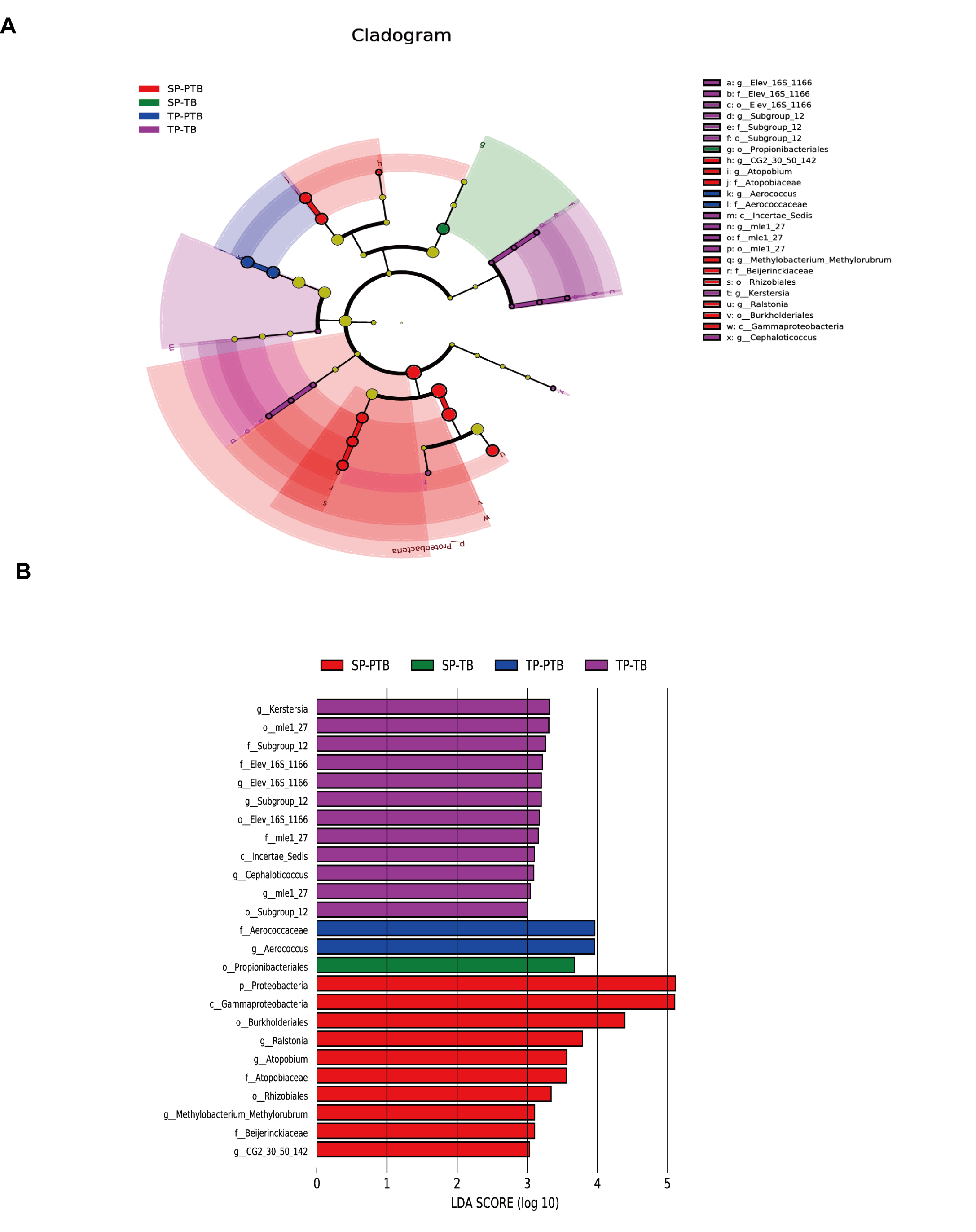
**

**Fig. S7** LEfSe analysis for comparing microbial variations in different groups at the species level. (A) LEfSe cladogram representing differentially abundant taxa (P < 0.05). (B) LDA scores as calculated by LEfSe of taxa are differentially abundant among groups. Only taxa with LDA scores of more than 2 are presented.

**
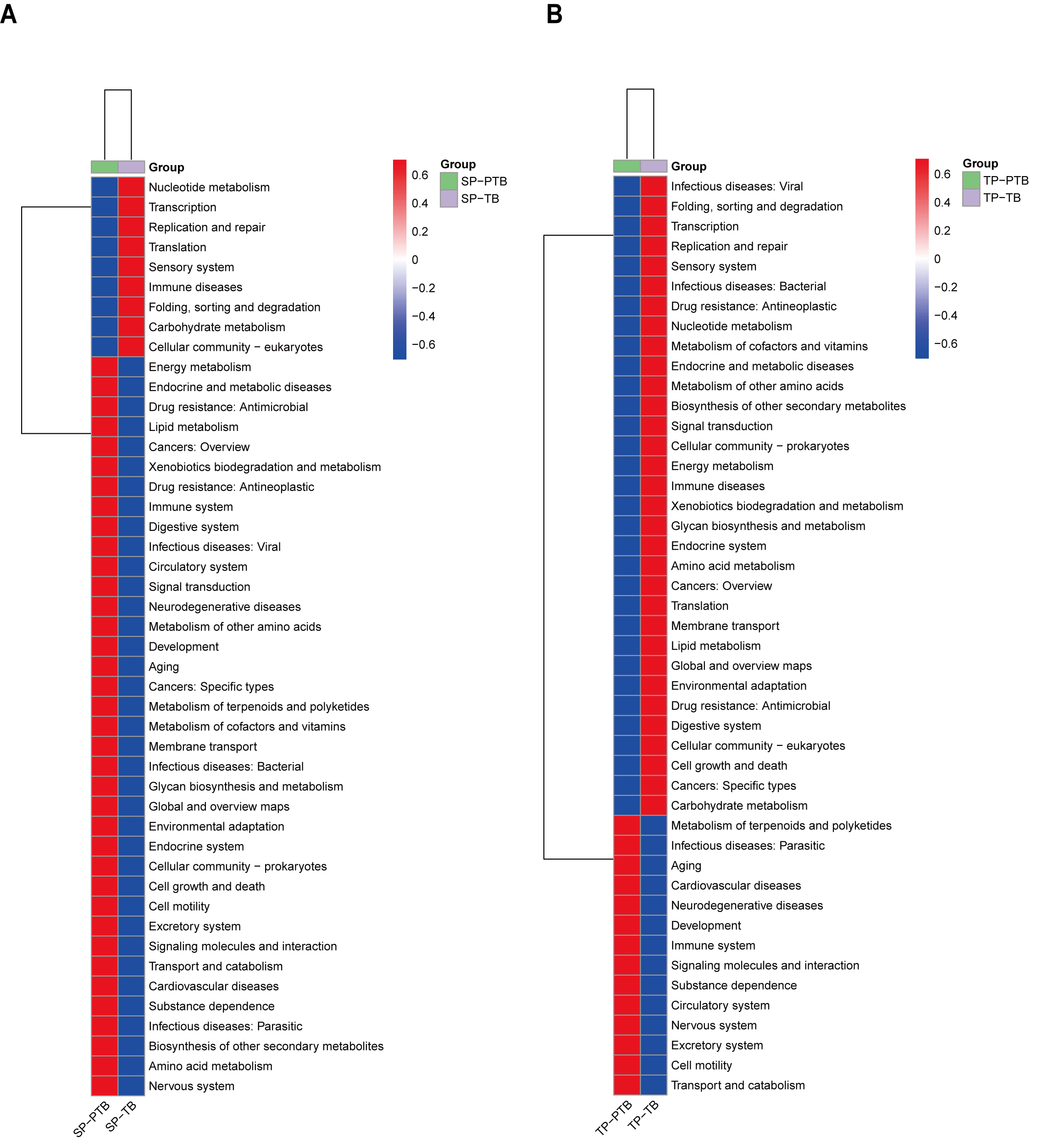
**

**Fig. S8 Functional prediction of cervicovaginal microorganisms by KEGG pathways.** KEGG, Kyoto encyclopedia of genes and genomes.


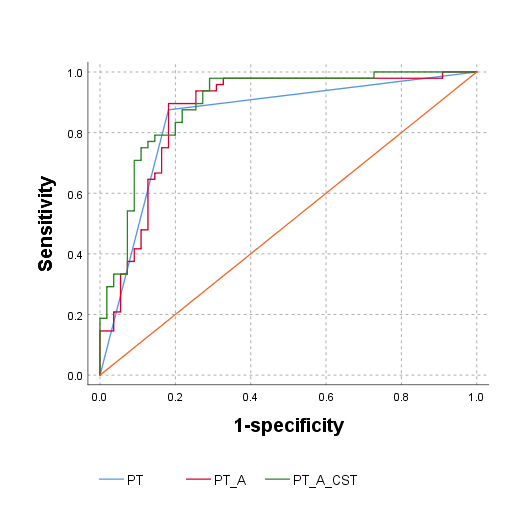

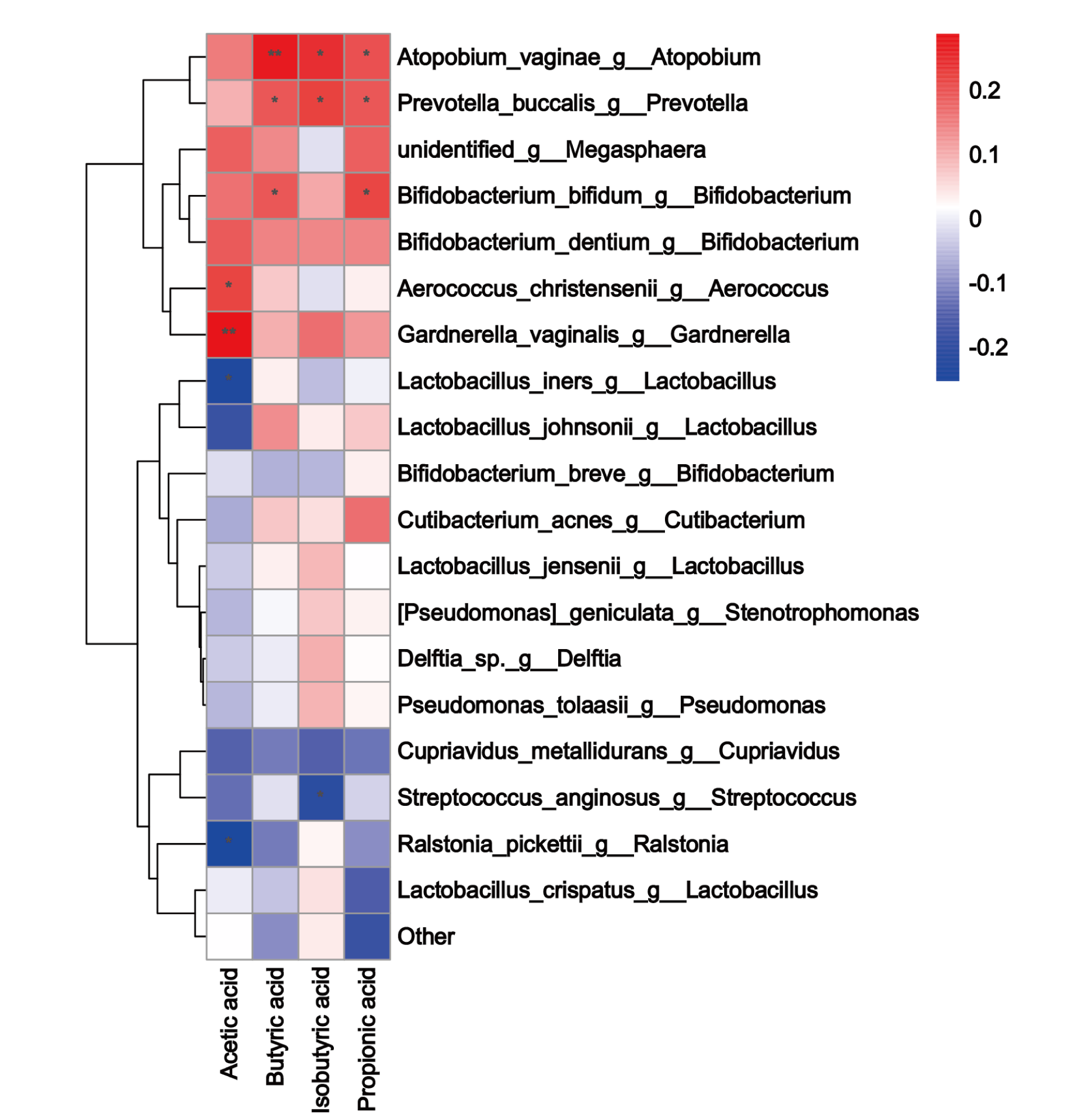
Fig.S9 Heatmap to show Spearman’s correlation between metabolites and microbiota composition at species level. Asterisk indicates a statistically significant correlation at the level of (*：*p*-value < 0.05；**：*p*-value < 0.01), and the color denotes positive (red) and negative (blue) correlation values.
