# Supplementary material for: Microbiome-producing SCFAs are associated with preterm birth via trophoblast function modulation
Source: mBio. 2024 Nov 11;15(12):e02702-24. doi: 10.1128/mbio.02702-24 (PMC11633107; doi:10.1128/mbio.02702-24)
Supplement: Supplemental tables — Tables S1 to S3. [file mbio.02702-24-s0002.docx]

**Supplementary Table 1. Western Blot Antibodies**

| **Antibody** | **Information** | **Dilution Ratio** |
| --- | --- | --- |
| anti-FFAR2 | 19952-1-AP | 1:2000 |
| anti-GAPDH | WB0197 | 1:2000 |
| anti-rabbit-IgG | WB0177 | 1:10000 |
| anti-mouse-IgG | WB0176 | 1:10000 |
| anti-pERK | CST4370 | 1:1000 |
| Anti-tERK | CST4695 | 1:1000 |

**Supplementary Table 2. Immunohistochemistry and Immunofluorescence Antibodies**

| **Antibody** | **Information** | **Dilution Ratio** |
| --- | --- | --- |
| anti-FFAR2 | 19952-1-AP  Ab124272 | 1:150 IHC  1:200 IF |
| anti-CK7 | 66483-1-Ig | 1:500 IHC |
| anti-HLA-G | Ab52454 | 1:200 IHC |
| anti-rabbit-IgG | Ab150077 | 1:500 IF |
| anti-mouse-IgG | Bs10027 | 1:500 IF |

**Supplementary Table 3. Changes in SCFA levels in the preterm birth group and term birth group**

| **Metabolites** | **PTB** | **TB** | **VIP** | ***P* value** | **Q-VALUE** | **FOLD CHANGE** | **LOG_FOLDCHANGE** |
| --- | --- | --- | --- | --- | --- | --- | --- |
| Acetic acid | 78.357 | 32.775 | 1.149 | 0.047^*^ | 0.030 | 2.391 | 1.257 |
| Propionic acid | 10.868 | 2.713 | 1.281 | 0.075 | 0.033 | 4.006 | 2.002 |
| Isobutyric acid | 2.544 | 0.936 | 1.042 | 0.244 | 0.062 | 2.717 | 1.442 |
| Butyric acid | 2.871 | 1.099 | 1.339 | 0.121 | 0.038 | 2.613 | 1.385 |
| Isovaleric acid | 3.598 | 0.625 | 0.990 | 0.310 | 0.072 | 5.759 | 2.526 |
| Valeric acid | 1.025 | 1.045 | 0.323 | 0.543 | 0.119 | 0.980 | -0.029 |
| Hexanoic acid | 1.827 | 1.989 | 0.755 | 0.101 | 0.036 | 0.918 | -0.123 |
| Heptanoic acid | 0.525 | 0.600 | 0.987 | 0.041^*^ | 0.030 | 0.874 | -0.194 |
| Octanoic acid | 0.794 | 0.928 | 0.983 | 0.055 | 0.030 | 0.856 | -0.225 |
| Nonanoic acid | 6.297 | 6.699 | 1.030 | 0.026^*^ | 0.030 | 0.940 | -0.089 |
| Decanoic acid | 0.386 | 0.436 | 0.710 | 0.286 | 0.068 | 0.886 | -0.174 |

The data represent the total and individual levels of short-chain fatty acids (SCFAs) measured in the cervicovaginal swabs.

Student's t-test; **p*<0.05 compared with baseline.
